# The response regulator Skn7 of *Aspergillus fumigatus* is essential for the antifungal effect of fludioxonil

**DOI:** 10.1038/s41598-021-84740-6

**Published:** 2021-03-05

**Authors:** Sebastian Schruefer, Isabella Böhmer, Karl Dichtl, Anja Spadinger, Christoph Kleinemeier, Frank Ebel

**Affiliations:** 1 Institute for Infectious Diseases and Zoonoses, Faculty of Veterinary Medicine, LMU Munich, Munich, Germany; 2 Max von Pettenkofer Institute, Faculty of Medicine, LMU Munich, Munich, Germany

**Keywords:** Microbiology, Molecular biology

## Abstract

*Aspergillus fumigatus* is an important fungal pathogen that represents a major threat for severely immunocompromised patients. Cases of invasive aspergillosis are associated with a high mortality rate, which reflects the limited treatment options that are currently available. The development of novel therapeutic approaches is therefore an urgent task. An interesting compound is fludioxonil, a derivative of the bacterial secondary metabolite pyrrolnitrin. Both agents possess potent antimicrobial activity against *A. fumigatus* and trigger a lethal activation of the group III hybrid histidine kinase TcsC, the major sensor kinase of the High Osmolarity Glycerol (HOG) pathway in *A. fumigatus*. In the current study, we have characterized proteins that operate downstream of TcsC and analyzed their roles in the antifungal activity of fludioxonil and in other stress situations. We found that the SskA-SakA axis of the HOG pathway and Skn7 can independently induce an increase of the internal glycerol concentration, but each of these individual responses amounts for only half of the level found in the wild type. The lethal fludioxonil-induced ballooning occurs in the *ssk*A and the *sak*A mutant, but not in the *skn*7-deficient strain, although all three strains show comparable glycerol responses. This indicates that an elevated osmotic pressure is necessary, but not sufficient and that a second, decisive and Skn7-dependent mechanism mediates the antifungal activity. We assume that fludioxonil triggers a reorganization in the fungal cell wall that reduces its rigidity, which in combination with the elevated osmotic pressure executes the lethal expansion of the fungal cells. Two findings link Skn7 to the cell wall of *A. fumigatus*: (1) the fludioxonil-induced massive increase in the chitin content depends on Skn7 and (2) the *skn*7 mutant is more resistant to the cell wall stressor Calcofluor white. In conclusion, our data suggest that the antifungal activity of fludioxonil in *A. fumigatus* relies on two distinct and synergistic processes: A high internal osmotic pressure and a weakened cell wall. The involvement of Skn7 in both processes most likely accounts for its particular importance in the antifungal activity of fludioxonil.

## Introduction

**Figure 1:**
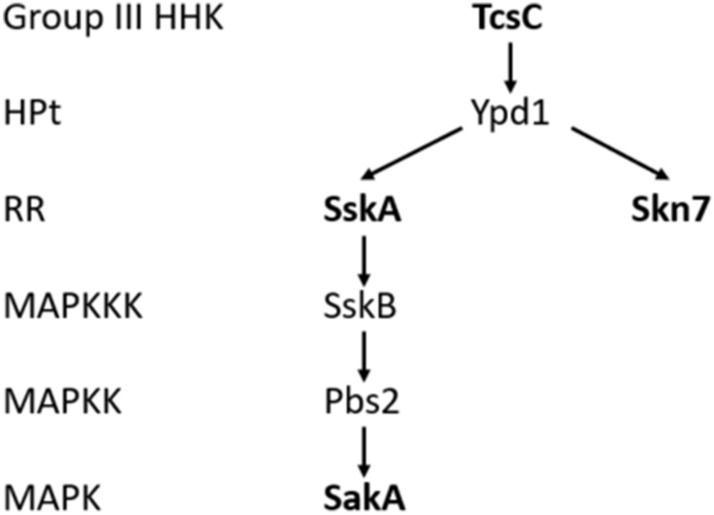
Schematic representation of the HOG pathway in *A. fumigatus*. Proteins that were characterized in this study are indicated in bold letters. Group III HHK = group III hybrid histidine kinase, HPt = histidine-containing phosphotransfer protein, RR = response regulator, MAPKKK = mitogen-activated protein kinase kinase kinase.

The High Osmolarity Glycerol (HOG) pathway is an important fungal signaling cascade that governs the cellular responses to several external stress situations, such as osmotic shock, UV irradiation, high temperature, oxidative and heavy metal stress^1^. In *Saccharomyces cerevisiae*, the histidine kinase Sln1 is a major sensor of the HOG pathway^2^, but in filamentous fungi, this function had been resumed by group III hybrid histidine kinases (HHK)^3^. The corresponding *A. fumigatus* protein was designated TcsC (for **T**wo **c**omponent **s**ystem **C**)^4^ and is part of a phospho-relay system that comprises TcsC, the phospho-transfer protein Ypd1 and the two response regulators SskA and Skn7. Skn7 is a transcription factor, whereas SskA relays signals to SskB, the first component of a MAP kinase cascade that terminates in SakA, the orthologue of *S. cerevisiae* Hog1 (see Fig. 1).

Group III HHKs have been characterized in several pathogenic fungi. Mutants in the corresponding genes are viable indicating that these proteins are not essentially required under normal lab conditions. Dependent on the respective pathogen, loss of the group III HHK may or may not result in an attenuated virulence (reviewed in^5^). The particular interest in the HOG pathway as a therapeutic target derives from the finding that a pharmacological activation of group III HHKs is often lethal for the fungus^6^. The corresponding natural lead substance is pyrrolnitrin that is produced by certain Gram-negative bacteria and possesses a pronounced antifungal activity^7^. Fludioxonil and fenpiclonil are synthetic derivatives of pyrrolnitrin that are more photo-stable and currently used as antifungal agents in agriculture to protect leaves, fruits and seeds at pre- and post-harvest stages. Even though these agents have now been utilized for decades, resistance in the field is rarely observed, although resistant mutants can be readily isolated under lab conditions^7^. These mutants commonly carry point mutations in their group III HHK, which apparently have a negative impact on their overall fitness^7^.

Several important fungal pathogens of humans are sensitive to fludioxonil; apart from *Cryptococcus neoformans*^8^ and *Candida albicans*^9^ this includes several pathogenic molds, e.g. *A. fumigatus*^10^. In *A. fumigatus,* fludioxonil triggers an immediate growth arrest, a reorganization of the cell wall and a dramatic and finally lethal expansion of the cells. The group III HHK TcsC is essentially required for the antifungal activity of fludioxonil and related agents^4,11^, but the molecular mechanisms that are activated by TcsC to execute the lethal effect are largely unknown. In this study, we have therefore analyzed several components of the HOG pathway that operate downstream of TcsC in order to investigate their relative importance in different stress situations and, in particular, to define their role in the antifungal activity of fludioxonil. Apart from the classical HOG pathway that runs from SskA to SakA, we have also included Skn7 in this analysis and consider this response regulator as a side branch of the HOG pathway.

## Results

In order to investigate the role of individual components of the *A. fumigatus* HOG pathway for the antifungal activity of fludioxonil, we have generated single deletion mutants in *ssk*A (Afu5g08390), *sak*A (Afu1g12940) and *skn*7 (Afu6g12522) as well as a double mutant in *skn*7 and *sak*A (Δ*skn*7 Δ*sak*A). A schematic representation of the HOG pathway in *A. fumigatus* is depicted in Fig. 1. All strains were verified by PCR analysis (Suppl. Fig. 1). On Aspergillus Minimal Medium (AMM), all strains including the already published Δ*tcs*C mutant^4^ and the parental strain AfS35 grew well and formed colonies of comparable size (Suppl. Fig. 2).

### The antifungal effect of fludioxonil requires Skn7

**Figure 2:**
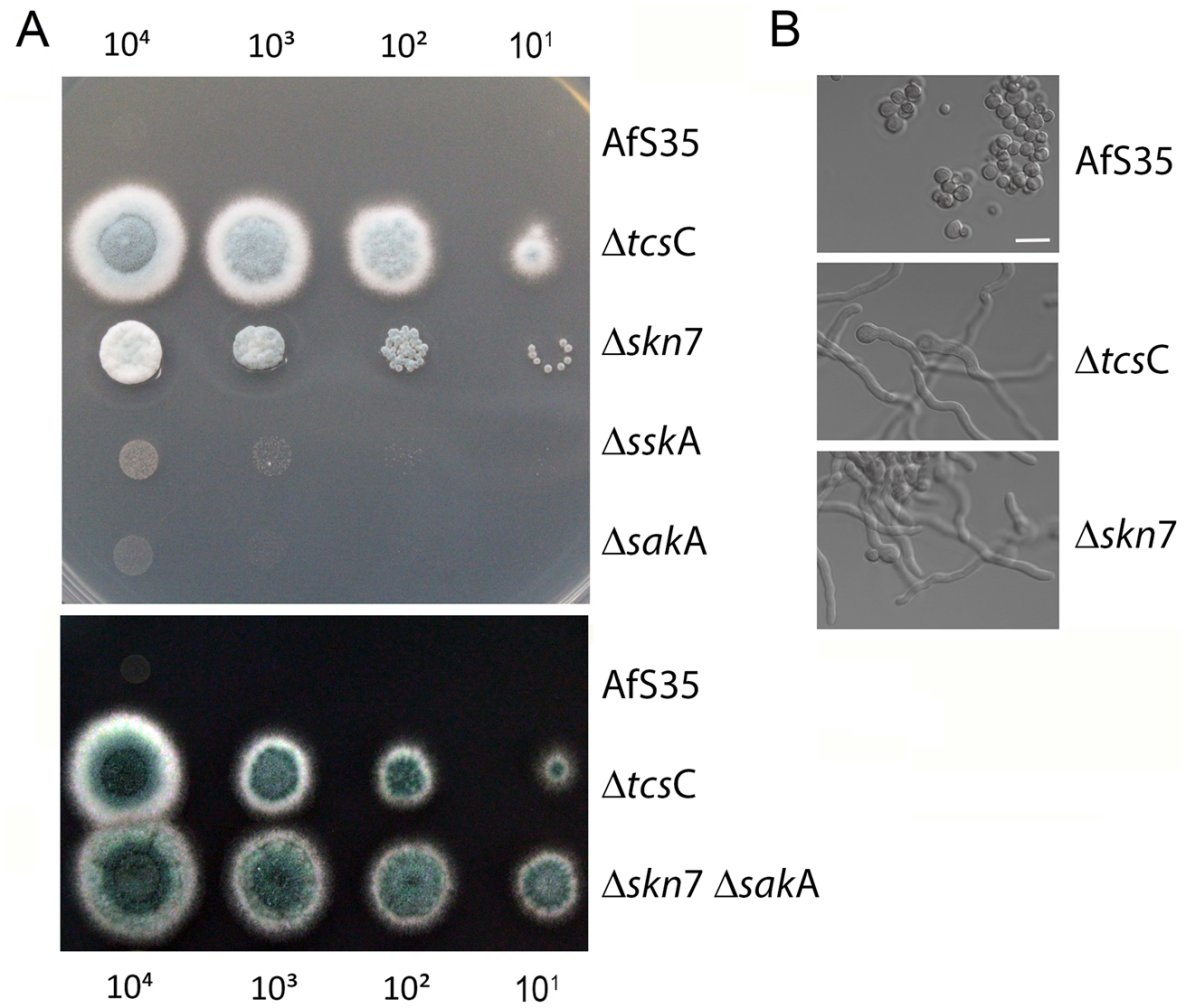
Growth of the HOG pathway mutants in the presence of fludioxonil. (Panel **A**) shows drop dilution assays on AMM plates containing 1 µg/ml fludioxonil. These plates were incubated for 48 h at 37 °C. The number of conidia per drop is indicated. (Panel **B**) shows bright field images of conidia of the indicated strains that were incubated for 20 h at 37 °C in AMM supplemented with 2 µg/ml fludioxonil. The bar in the upper image represents 10 µm and is valid for all images.

Fludioxonil is assumed to trigger a permanent and lethal activation of group III HHKs^6^. In *A. fumigatus*, this results in a massive swelling, an accumulation of large numbers of nuclei, an enhanced septation and changes in the cell wall composition^10,12^. From all strains tested in drop dilution assays in the presence of fludioxonil (1 µg/ml), only the Δ*tcs*C and the Δ*skn*7 Δ*sak*A double mutant were strongly resistant (Fig. 2A,B). The Δ*ssk*A*,* the Δ*sak*A mutant and their parental strain AfS35 were highly sensitive and the Δ*skn*7 strain showed a partial resistance (Fig. 2A). The resistant phenotype of the Δ*skn*7 mutant was even more pronounced in a disk diffusion assay and in liquid medium (see below), assays in which the growth of Δ*skn*7 resembled that of Δ*tcs*C. We have measured an identical minimal inhibitory concentration of 0.3 µg fludioxonil/ml for the sensitive strains AfS35, Δ*ssk*A and Δ*sak*A in minimal medium (AMM). Under the same assay conditions, the resistant strains Δ*tcs*C, Δ*skn*7 and Δ*skn*7 Δ*sak*A grew even in the presence of 20 µg/ml, the highest concentration tested at which fludioxonil was already partially insoluble. The different strains showed similar patterns of sensitivity to the fludioxonil-related compounds pyrrolnitrin and fenpiclonil (Suppl. Fig. 3), demonstrating that the antifungal activities of all three agents on *A. fumigatus* are largely dependent on Skn7, whereas SskA and SakA are only of minor importance.

### Fludioxonil treatment results in elevated cytoplasmic glycerol concentrations

**Figure 3:**
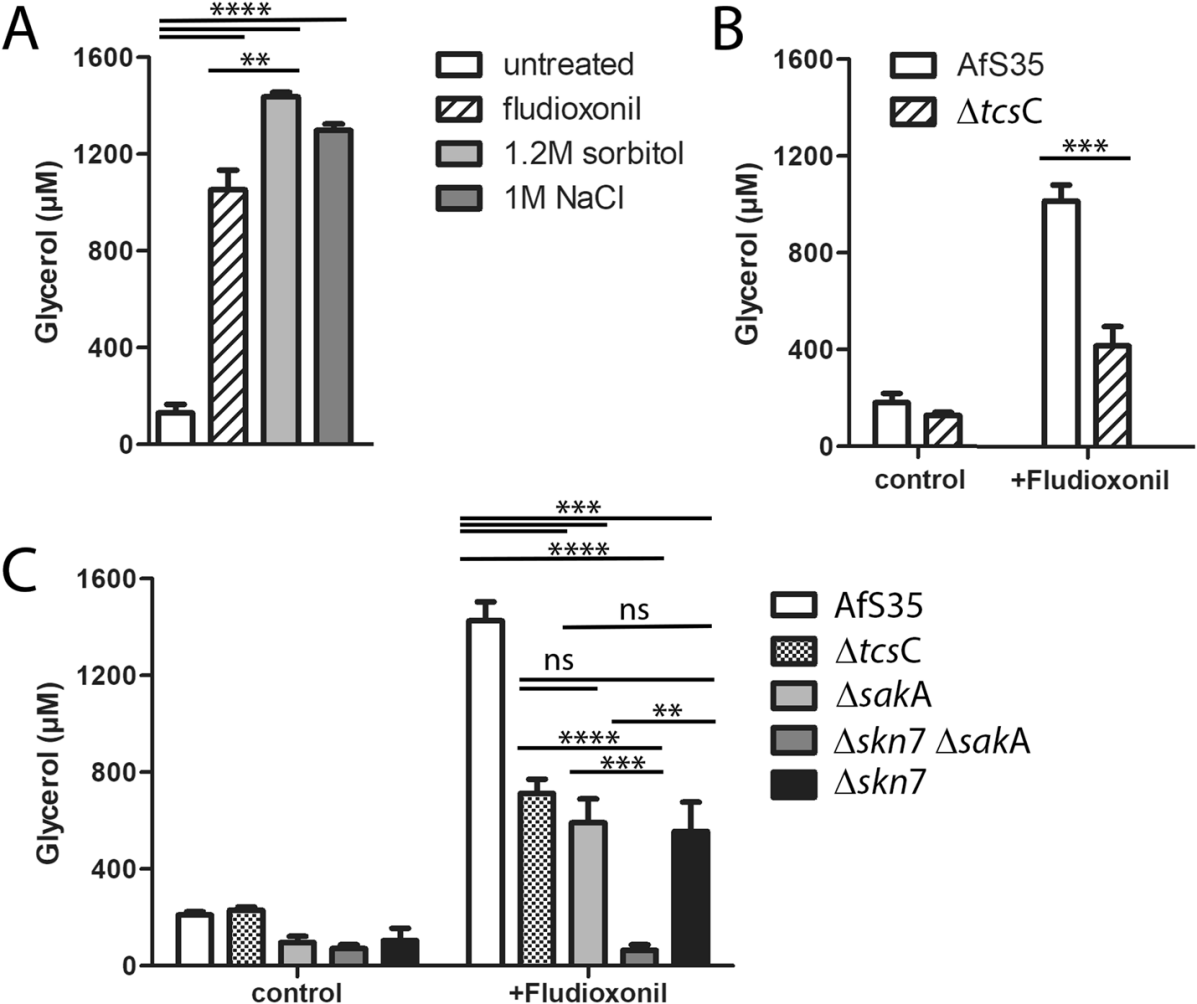
Changes of the intracellular glycerol concentration in response to different stress situations. (Panel **A**) The *A. fumigatus* wild type responded to treatments with fludioxonil (1 µg/ml), 1.2 M sorbitol or 1 M NaCl with a similar increase of its intracellular glycerol concentration. (Panel **B** and **C**) Fludioxonil (1 µg/ml) triggered a moderate increase in the intracellular glycerol concentrations of the Δ*tcs*C, Δ*skn*7, Δ*sak*A and Δ*ssk*A mutants, whereas the Δ*skn*7 Δ*sak*A mutant was non-responsive. Hyphae were pre-grown for 16 h at 30 °C. After addition of the stressor the incubation was prolonged for 6 h at 37 °C. The glycerol concentrations were determined for hyphal extracts adjusted to a protein concentration of 100 µg/ml. Data were analyzed using a Student’s t-test (**: *p* < 0.01, ***: *p* < 0.001, ****: *p* < 0.0001).

Fludioxonil triggered a strong increase in the cytoplasmic glycerol concentration of the *A. fumigatus* wild type strain AfS35, which was comparable to those induced by 1.2 M sorbitol or 1 M NaCl (Fig. 3A). Compared to wild type, the response of the Δ*tcs*C mutant was weaker, but clear-cut (Fig. 3B). This phenotype was unexpected, but reproducible in several experiments and with different, independently generated Δ*tcs*C mutant strains (data not shown). Further experiments showed that the Δ*skn*7 Δ*sak*A double mutant was the only non-responsive strain indicating that an increase of the internal glycerol concentration requires either Skn7 or the SskA-SakA axis (Fig. 3C). The Δ*skn*7, Δ*sskA* and Δ*sak*A single mutants responded to fludioxonil with a comparable increase in their glycerol concentration that amounted to approximately half of that found in the wild type (Fig. 3C).

### Characterization of the HOG mutants under hyperosmotic stress

**Figure 4:**
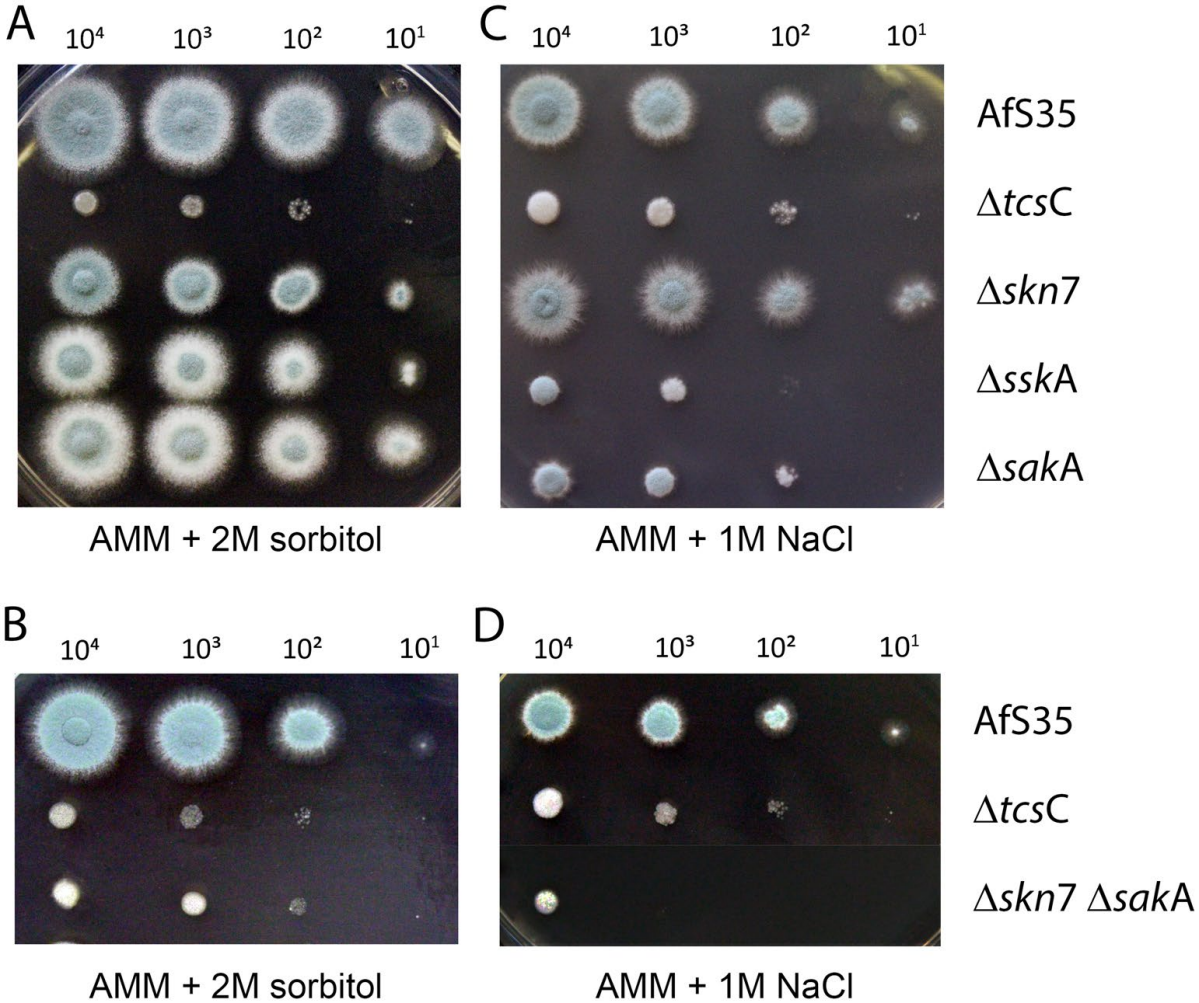
Impact of hyperosmotic stress on the growth of *A. fumigatus* HOG pathway mutants. Drop dilution assays on plates containing AMM + 2 M sorbitol (panels **A** and **B**) and AMM + 1 M NaCl (panels **C** and **D**). Plates were incubated at 37 °C for 72 h. The strains and the number of conidia that were applied per drop are indicated.

Since Skn7 and the SskA-SakA axis trigger a similar and independent glycerol increase in response to fludioxonil, we next analyzed their relevance in the adaption to hyperosmotic stress. On AMM plates containing either 1.2 M or 2 M sorbitol, only the Δ*tcs*C and the Δ*skn*7 Δ*sak*A double mutant were severely impaired in growth (Fig. 4A,B and data not shown), whereas high salt concentrations (1 M NaCl and 1 M KCl) also reduced the growth of the Δ*sak*A and Δ*ssk*A strains (Fig. 4C,D and data not shown). This confirms the previous observation that TcsC is essentially required for the adaptive responses to high salt and elevated sorbitol concentrations^4,11^. In the case of high salt stress, adaption requires the activities of SskA and SakA. Taken together, our data indicate that both proteins mediate a glycerol increase in response to fludioxonil (Fig. 3C), but are not essential for the adaptation to high sorbitol. The growth inhibition of the Δ*sak*A mutant in the presence of high salt was instrumental to demonstrate its functional complementation (Suppl. Fig. 4B).

### Skn7 is a key player in the response of *A. fumigatus* to oxidative stress

**Figure 5:**
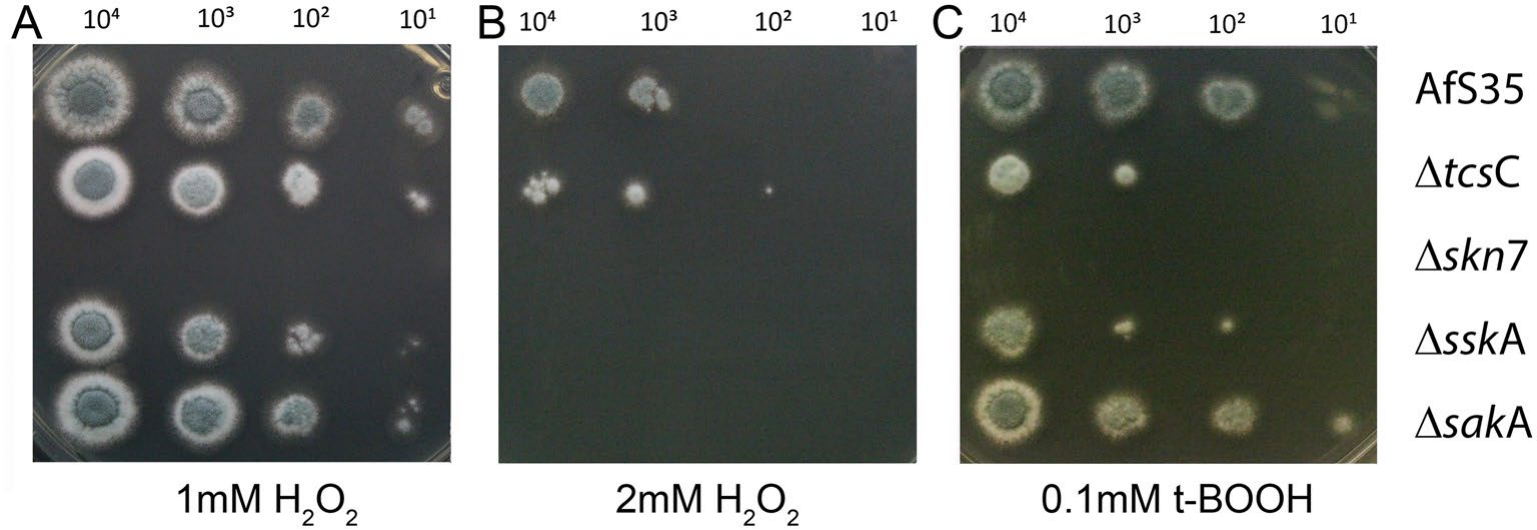
Growth of the HOG pathway mutants under oxidative stress conditions. Drop dilution assays on AMM plates containing 1 mM H_2_O_2_ (panel **A**), 2 mM H_2_O_2_ (panel **B**) or 0.1 mM t-BOOH (panel **C**) were incubated for 48 h at 37 °C. The strains and the number of conidia that were applied per drop are indicated.

It was already shown in a previous study, that an *A. fumigatus* mutant lacking *skn*7 was highly sensitive to oxidative stress^13^. Accordingly, we found our Δ*skn*7 strain to be also highly sensitive to 1 mM H_2_O_2_ (Fig. 5A) and 0.1 mM tert-butyl hydroperoxide (tBOOH) (Fig. 5C). The other single mutants were clearly less sensitive and the ∆*sak*A mutant showed a wild type-like resistance. This pattern changed in the presence of 2 mM H_2_O_2_, here the ∆*ssk*A, the ∆*sak*A and to a lesser degree also the ∆*tcs*C mutant were clearly more sensitive than the wild type (Fig. 5B). The phenotype of the Δ*skn*7 Δ*sak*A double mutant resembled that of the Δ*skn*7 single mutant in these assays (data not shown). Expression of *skn*7 from its native promoter fully restored wild type-like phenotypes on plates containing 1 mM H_2_O_2_ or 1 µg/ml fludioxonil (Suppl. Fig. 4C,D).

The particular importance of Skn7 for the antifungal activity of fludioxonil and the response to oxidative stress raised the possibility that the impact of fludioxonil may rely on the generation of reactive oxygen species. To address this point, we used 2′,7′-dichlorofluorescin diacetate, a cell-permeable probe that becomes fluorescent upon oxidation, but observed no increased fluorescence after treatment with fludioxonil (data not shown).

### The Δskn7 mutant shows an enhanced resistance to Calcofluor white

**Figure 6:**
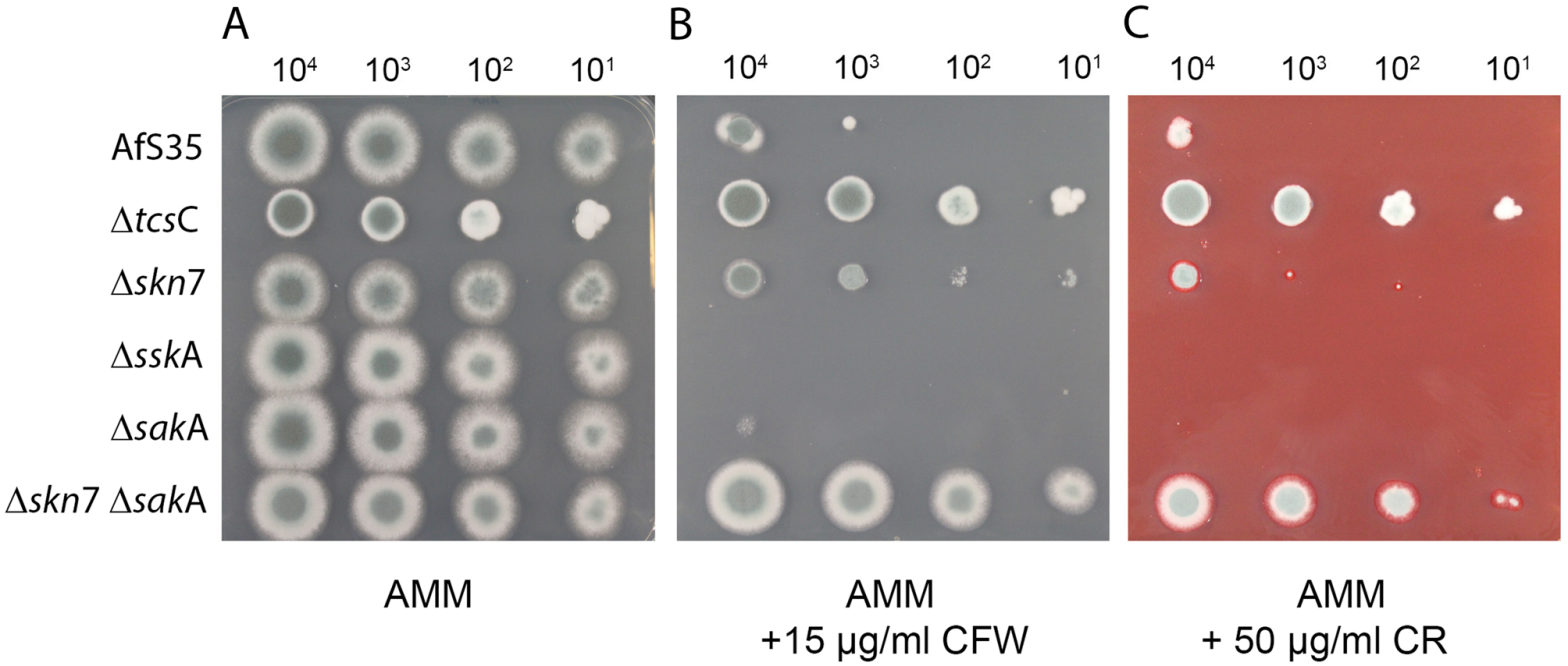
Growth of the HOG mutants in the presence of cell wall stressors. Drop dilution assays on AMM plates containing no stressor (panel **A**), 15 µg/ml Calcofluor white (panel **B**) or 50 µg/ml Congo red (panel **C**). The plates were incubated for 48 h at 37 °C. The number of conidia applied per drop is indicated.

The HOG pathway has also been implicated in the adaption to cell wall stress. In this study, we have used the well-known cell wall stressors Congo red (CR) and Calcofluor white (CFW). A control plate without stressors is shown in Fig. 6A. The Δ*tcs*C mutant showed a strikingly elevated resistance to both compounds (Fig. 6B,C), which is in line with previously published data^4,11^. A similar phenotype was observed for the Δ*skn*7 Δ*sak*A double mutant, whereas the Δ*sskA* and Δ*sak*A single mutants were even more sensitive than the wild type (Fig. 6B,C). The Δ*skn*7 mutant had a wild type-like resistance to CR (Fig. 6C). In the presence of CFW it was more resistant than the wild type and more sensitive than the Δ*tcs*C and Δ*skn*7 Δ*sak*A mutants (Fig. 6B). This suggests that the cell wall-damaging activity of CFW involves TcsC and Skn7, but the different phenotypes of the Δ*skn*7 and the Δ*skn*7 Δ*sak*A double mutant also indicate a relevance of SakA in this context.

We have furthermore compared the sensitivity of the different strains to caspofungin, an antifungal that inhibits the synthesis of β-1,3-glucan^14^. Using a disk diffusion assay we found comparable inhibition zones for all strains after 24 h. However, after prolonged incubation, the wild type started to grow and sporulate within the inhibition zone, whereas the Δ*tcs*C, the Δ*skn*7, Δ*sak*A and the double mutant were all impaired in this so-called trailing growth and showed a clearly reduced sporulation compared to the wild type (Suppl. Fig. 5). This indicates that the adaptive processes that permit growth in the presence of high caspofungin concentrations involve TcsC and its downstream proteins.

### Skn7 is required for the fludioxonil-induced increase in the chitin content of the cell wall

**Figure 7:**
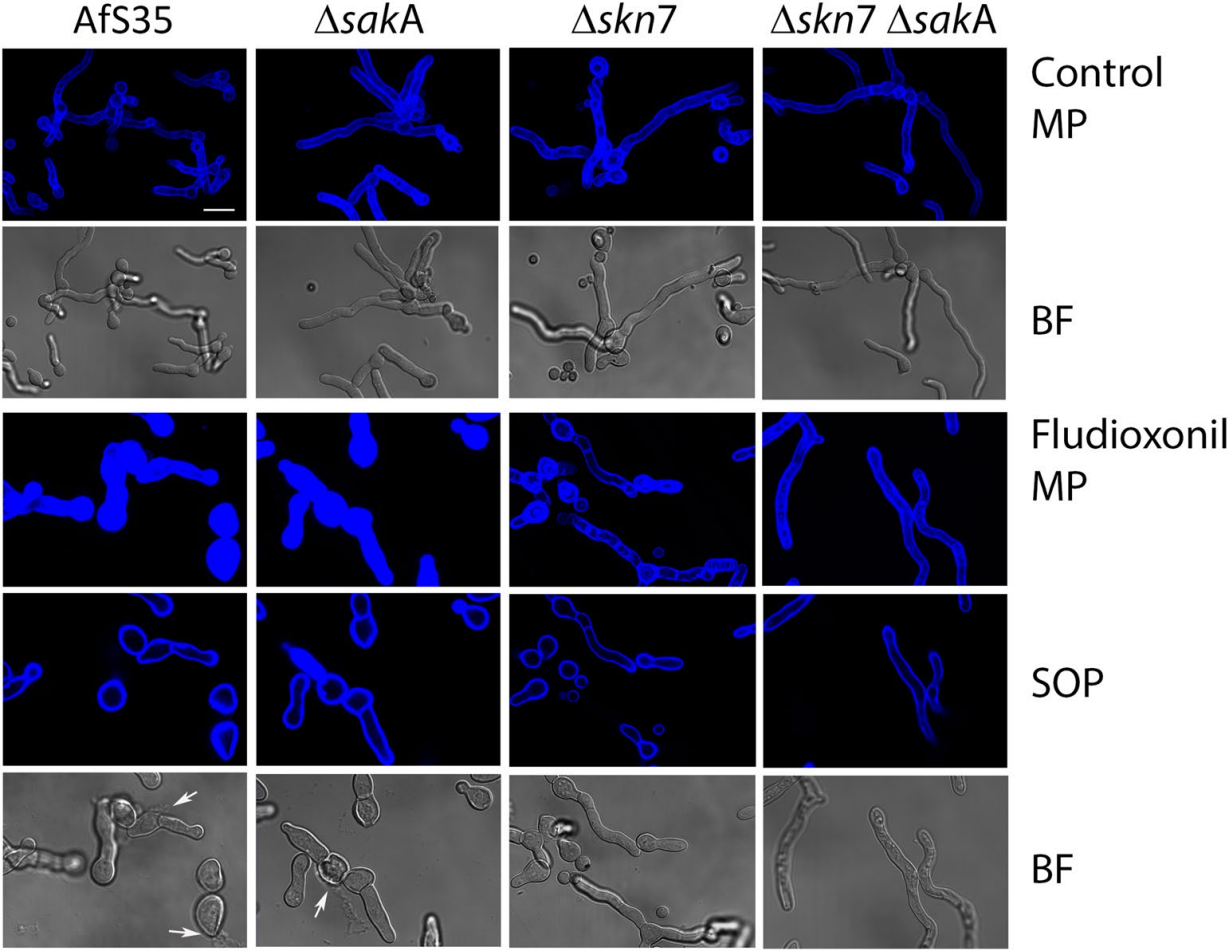
Analysis of fludioxonil-induced changes of the chitin content in the cell wall. Resting conidia of the indicated strains were cultivated in AMM with or without fludioxonil (1 µg/ml) for 16 h at 37 °C. Samples were fixed and stained with CFW, the corresponding bright field images were also shown. MP = maximum intensity projections of z-stacks. SOP = Single optical planes of the corresponding z-stack. BF = bright field image. Lysing cells are indicated by arrows. The bar in the upper left panel represents 10 µm and is valid for all panels.

Resting conidia of the fludioxonil-sensitive strains AfS35 (wild type) and Δ*sak*A as well as the resistant strains Δ*skn*7 and Δ*skn*7 Δ*sak*A were grown in AMM supplemented with 1 µg/ml fludioxonil. After 16 h at 37 °C the samples were fixed and stained with the chitin-specific dye CFW. Fludioxonil treatment arrested the two sensitive strains in an early stage of germination. The resulting germlings showed signs of ballooning, lysis and an extremely strong chitin staining (Fig. 7). Similar results were also obtained for the *ssk*A mutant (data not shown). The ∆*skn*7 and the ∆*skn*7 ∆*sak*A double mutant were, in contrast, able to form hyphae with a normal morphology and showed a chitin staining that resembled that of control hyphae grown without fludioxonil (Fig. 7).

### During germination, *A. fumigatus* is particular susceptible to fludioxonil

**Figure 8:**
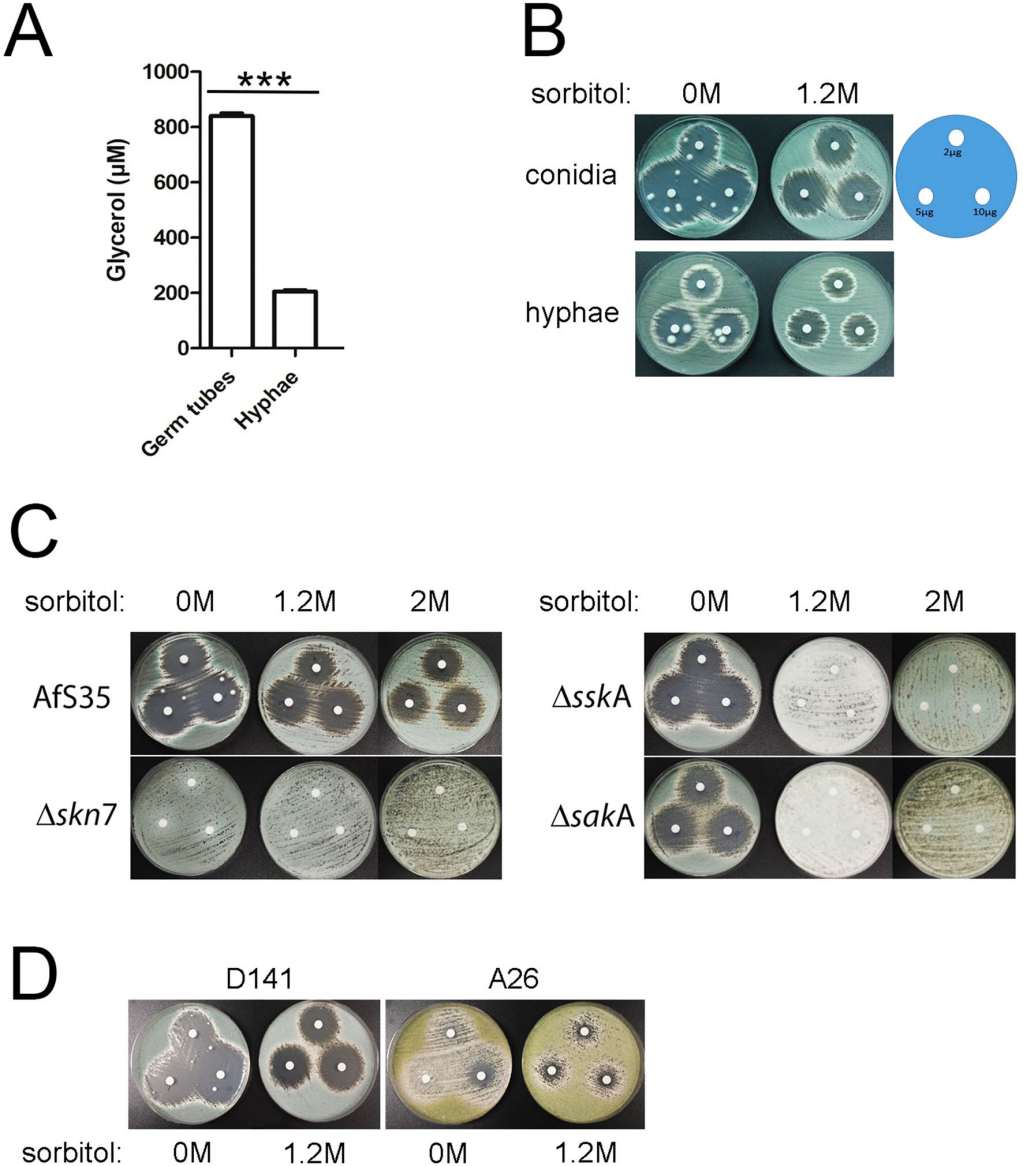
Impact of osmoprotection on the antifungal activity of fludioxonil. (Panel **A)** comparison of the glycerol concentrations of extracts obtained from germ tubes and hyphae of *A. fumigatus*. The data were analyzed using a Student’s t-test, ***: *p* < 0.001. (**B**) Fludioxonil loaded paper disks were placed on AMM plates with resting conidia (conidia) or pre-grown hyphae (hyphae) that were in part supplemented with 1.2 M sorbitol. The disks contained 2, 5 and 10 µg of fludioxonil as indicated. Note that resistant colonies in the inhibition zones occurred only on plates without sorbitol. (Panel **C**) shows a similar osmoprotection experiment as in (panel **B**), but this time we compared the wild type and different HOG mutants. In this experiment, all plates were inoculated with resting conidia and contained either 0 M, 1.2 M or 2 M sorbitol as indicated. The paper disks contained 2, 5 and 10 µg of fludioxonil and were placed as indicated in (panel **B**). (Panel **D**) shows a comparison of *A. fumigatus* strain D141 and the *A. nidulans* strain A26. Plates were inoculated with resting conidia. The different sorbitol concentrations are indicated. The paper disks contained 2, 5 and 10 µg of fludioxonil and were placed as indicated in (panel **B**).

The elevated osmotic pressure in fludioxonil-treated cells is assumed to drive the characteristic ballooning. We therefore hypothesized that the impact of fludioxonil is largely determined by the difference between the internal and external osmotic pressure. The germination process is associated with a transient increase of the internal osmotic pressure that drives the isotropic growth phase. Measurements of the glycerol concentrations in early germ tubes and hyphae indeed revealed that the concentrations in germ tubes were clearly higher than those in hyphae (Fig. 8A). Remarkably, the concentrations measured for germ tubes were comparable to those found in fludioxonil-treated hyphae (compare Fig. 3). We therefore expected differences in the fludioxonil sensitivity at different stages of the germination process. To investigate this, we performed two experiments: In the first, we inoculated pairs of AMM plates with resting conidia of strain AfS35. One plate was stored at 4 °C and the other was pre-grown to the stage of short hyphae. Paper disks were then placed on both plates and loaded with fludioxonil. After another 48 h at 37 °C, the inhibition zones were clearly larger for those plates that were not pre-grown (Fig. 8B) indicating that the susceptibility of *A. fumigatus* is particularly high during germination. In the second experiment, we tested a potentially protective effect of 1.2 M sorbitol in the medium. We indeed obtained evidence for osmoprotection and this was particular prominent on plates starting at the stage of resting conidia and indicated by a smaller inhibition zone (Fig. 8B, upper panel). Strikingly, spontaneous resistant mutant colonies appeared on normal AMM plates, but not on those containing 1.2 M sorbitol. This indicates that the resistance to fludioxonil is accompanied by an enhanced sensitivity to hyperosmotic stress.

### The lethal effect of fludioxonil can be overcome by osmoprotection, but only in strains lacking *sskA* or *sakA*

We compared the different mutants on plates that were homogenously inoculated with resting conidia and contained 1.2 M, 2 M or no sorbitol. The Δ*tcs*C and the Δ*skn*7 Δ*sak*A mutants were, as expected, resistant to fludioxonil (data not shown). The Δ*skn*7 strain also grew on the whole plates indicating its resistance to fludioxonil. Inhibition zones were clearly detectable for Δ*sskA*, Δ*sak*A and the parental strain AfS35 on the control plates without osmoprotection. The presence of high sorbitol concentrations reduced the inhibition zones of the wild type strain and this effect was stronger for 2 M sorbitol. Strikingly, the Δ*ssk*A and the Δ*sak*A mutant showed wild type-like inhibition zones on the control plates, but were completely protected by both sorbitol concentrations (Fig. 8C).

We regularly observed few spontaneously resistant colonies of *A. fumigatus* in the fludioxonil inhibition zones (Fig. 8B–D), but such colonies did not occur on plates containing sorbitol (Fig. 8C). Moreover, such resistant colonies were also not detectable for the Δ*sskA* and Δ*sak*A mutant (on plates without sorbitol) (Fig. 8B,C). This suggests that a functional SskA-SakA axis is a prerequisite for these spontaneous mutants.

We have previously compared the impact of fludioxonil on *A. fumigatus* and *A. nidulans* and found the latter to be less sensitive^10,12^. A comparison of *A. nidulans* strain A26 and the *A. fumigatus* strain D141 again showed that the inhibition zones of *A. nidulans* were clearly smaller (Fig. 8D). Addition of 1.2 M sorbitol slightly reduced the inhibition zone of strain D141, but nearly abrogated that of A26 (Fig. 8D). Further tests with additional *A. fumigatus* (ATCC46645 and Af293) and *A. nidulans* strains (R21 and A4) showed that this difference is species- and not strain-specific (Suppl. Fig. 6).

## Discussion

The damaging activity of fludioxonil is mediated by interference with the fungal two-component signaling, a conserved sensing mechanism that enables fungi to adapt to various stress situations and environmental changes^1^. The mode of action employed by fludioxonil is not yet fully understood, but a crucial step is the activation of a group III HHK^6^. The resulting misdirected activation of the HOG pathway entails a dramatic imbalance between the internal and external osmotic pressure and consequently a dramatic expansion of the cellular body up to the point of lysis. Hence, the important physiologic role of group III HHKs to maintain an osmotic homeostasis is harnessed by fludioxonil and related compounds in order to damage the fungus.

Yeast cells can sense osmotic stress by several, redundant mechanisms involving distinct membrane proteins. One option is that high external osmolarity activates the HOG pathway through the inactivation of Sln1, a membrane histidine kinase that responds to changes in turgor pressure^15^. Adaptation is achieved by accumulation of intracellular glycerol, a process that is also governed by the HOG pathway^16^. Glycerol is a compatible osmolyte, meaning that its concentrations can be increased to restore the osmotic equilibrium and this without impairment of other cellular functions.

In this study, we found that fludioxonil triggers a strong increase of the cytoplasmic glycerol concentration of *A. fumigatus.* The resulting levels are in the same range as those induced by hyperosmotic stress (1.2 M sorbitol or 1 M NaCl). The glycerol responses in the different HOG single mutants were comparable and amounted for approximately half of that of the wild type. The ∆*skn*7 ∆*sak*A double mutant was the only strain that was non-responsive to fludioxonil. These data indicate that Skn7 and the SskA-SakA axis can both and independently cause an increase of the osmotic pressure. Both responses are additive and required to accomplish the wild type level. Such a moderate fludioxonil-induced glycerol increase was also detected in the resistant Δ*tcs*C mutant. This finding was unexpected and indicates that (1) an elevated osmotic pressure in the range of 50% of the wild type level is not sufficient to provoke significant damage and that (2) the fludioxonil-induced increase of the osmotic pressure in the wild type is in part independent of TcsC. Moreover, it needs to be stressed in this context that the TcsC-independent glycerol increase is either not triggered by hyperosmotic stress or is not sufficient to allow a successful adaptation, which is evidenced by the fact that the Δ*tcs*C mutant is unable to grow in the presence of 1.2 M sorbitol or 1 M NaCl.

According to our data, the sensitivity of *A. fumigatus* to fludioxonil is particularly pronounced during germination. Two factors may contribute to this phenotype: (1) the elevated internal osmotic pressure that drives the initially isotropic growth of conidia and (2) the more flexible organization of the conidial cell wall that allows a stretching or unfolding, which is in turn required for the fast increase of the cellular surface that is a characteristic feature of this growth phase.

Several results of the current study challenge the concept that the antifungal activity of fludioxonil is exclusively caused by a high internal osmotic pressure. First, all single mutants showed a similar and only moderate increase of their glycerol contents, but the impact of fludioxonil on these strains differs dramatically: The Δ*ssk*A and Δ*sak*A are highly sensitive, with a wild type-like ballooning and a severe growth inhibition, whereas the Δ*skn*7 and the Δ*tcs*C mutant are fludioxonil-resistant and show no dramatic swelling. Taken together, this hints to another fludioxonil-induced activity that determines the antifungal effect and essentially involves Skn7.

Skn7 is a highly conserved stress-responsive transcription factor and apart from Ssk1/SskA the second response regulator that can be activated via the phospho-transfer protein Ypd1. Skn7 plays a well-established role in the oxidative stress response and our data corroborate this for *A. fumigatus*. However, Skn7 was also shown to be involved in maintenance of the cell wall integrity of *S. cerevisiae* and other fungi^17–21^. It was in fact initially identified as a gene whose over-expression inhibits cell wall defects caused by mutations of the cell wall synthesis gene KRE9^22^. Baker’s yeast lacks a group III HHK and instead harbours the membrane-bound, osmosensing histidine kinase Sln1 that also relays its signals via Ypd1. Several studies indicate that Sln1 governs some, but not all Skn7 activities^17^ indicating that, at least in *S. cerevisiae,* Skn7 receives signals from distinct upstream sensor proteins.

Several studies have already implicated Skn7 in the antifungal activity of fludioxonil, e.g. in *Cryptococcus neoformans*^8^ and *Cochliobolus heterostrophus*^23^. In *A. nidulans*, the double mutant lacking SskA and SrrA (the orthologue of Skn7) was fully resistant to fludioxonil, whereas both single mutants showed a wild type-like sensitivity^24,25^. However, Hagiwara et al.^24^ noted that the fludioxonil sensitivity of the ∆*srr*A mutant was only detectable on solid medium, whereas the same strain was resistant in liquid culture. This resembles our finding that the resistance of the Δ*skn*7 mutant is particular pronounced in liquid culture. In the plant pathogen *Botrytis cinerea*, Liu et al.^26^ showed that the effect of fludioxonil and related antifungals depends on the group III HHK Bos1, but is largely independent of SakA. Hence, several lines of evidence implicate Skn7 in the antifungal mechanisms triggered by fludioxonil, but the underlying mechanisms remain obscure.

It is generally accepted that Skn7 is particular important in the response to oxidative stress and this is also true for *A. fumigatus*. We initially assumed that fludioxonil may damage *A. fumigatus* by oxidative stress, since fludioxonil was previously reported to trigger an enhanced expression of the hyphal catalase A gene^11^. We used 2′,7′-dichlorofluorescin diacetate to detect elevated levels of reactive oxygen species, but found no evidence that fludioxonil triggers an oxidative stress (our unpublished data).

The dramatic swelling is the hallmark of the antifungal activity of fludioxonil and this process is driven by an elevated internal osmotic pressure. The complete resistance of the Δ*skn*7 Δ*sak*A double mutant correlates well with the inability of fludioxonil to increase the glycerol concentration in this strain. The Δ*tcs*C and Δ*skn*7 mutants are also resistant, but their glycerol concentrations increase moderately in response to fludioxonil. Since comparable increases were also found in the fludioxonil-sensitive strains Δ*ssk*A and Δ*sak*A, it is apparent that Δ*tcs*C and Δ*skn*7 lack an important aspect of the fludioxonil-induced activity. Thus, our data imply that a certain increase of the internal pressure is a prerequisite for the fludioxonil-triggered cellular swelling, but another mechanism is also required and largely determining the outcome. All fludioxonil-sensitive strains possess Skn7 suggesting that this response regulator is essentially involved. Skn7 has been implicated in the cell wall organization and the rigidity of the cell wall counteracts and limits any expansion of the cellular volume. Hence, a Skn7-mediated weakening of the cell wall stability could reinforce the impact of the elevated internal pressure.

Fludioxonil causes an increase of the chitin content in the *A. fumigatus* cell wall^10^. In this study, we found that this characteristic increase of the chitin content does not occur in the ∆*skn*7 mutant and we observed a higher resistance of the ∆*skn*7 mutant to the cell wall stressor CFW. Both findings suggest a different cell wall structure in this mutant and implicate Skn7 in the fludioxonil-dependent cell wall reorganizations*.* Based on these data, we propose that the characteristic ballooning of fludioxonil-treated cells results from two parallel and synergistic processes: An increased internal osmotic pressure and a reduced rigidity of the cell wall. The SskA-SakA axis is only important for the osmotic response, whereas Skn7 seems to be also involved in the cell wall reorganizations and this may explain the particular resistance of the ∆*skn*7 mutant to fludioxonil and related antifungal compounds.

This model is also supported by osmoprotection experiments that revealed three phenotypes: (1) fludioxonil resistance in the absence of osmoprotection for Δ*tcs*C, Δ*skn*7 and Δ*skn*7 Δ*sak*A, (2) resistance upon osmoprotection for Δ*ssk*A and Δ*sak*A and (3) a limited osmoprotection for the wild type. Thus, only strains that possess Skn7 and TcsC, but lack either SskA or SakA can be efficiently protected. According to our model, the first group is resistant because its members lack the important contribution of Skn7 to the antifungal activity of fludioxonil; the second group develops a less rigid cell wall after fludioxonil treatment, which renders these strains in principle sensitive to fludioxonil, but the internal osmotic pressure is only moderately increased and can be compensated by a hyperosmotic medium. In the wild type, the induced cell wall instability and the high internal osmotic pressure act synergistically and even a medium containing 2 M sorbitol is not protective. Hence, the unique feature of the wild type is that fludioxonil can activate both Skn7 and the SskA-SakA axis and their concerted activities results in a particular robust antifungal effect that is largely resistant to osmoprotection.

Another interesting finding is that *A. nidulans* is less sensitive to fludioxonil than *A. fumigatus.* In light of the data obtained in this study, we assume that the wiring of Skn7 to processes that control the cell wall architecture differs in these two species. This could explain why some phenotypes of the *A. fumigatus* HOG mutants are similar, but not identical to those reported for their *A. nidulans* orthologues^24,25,27,28^.

**Figure 9:**
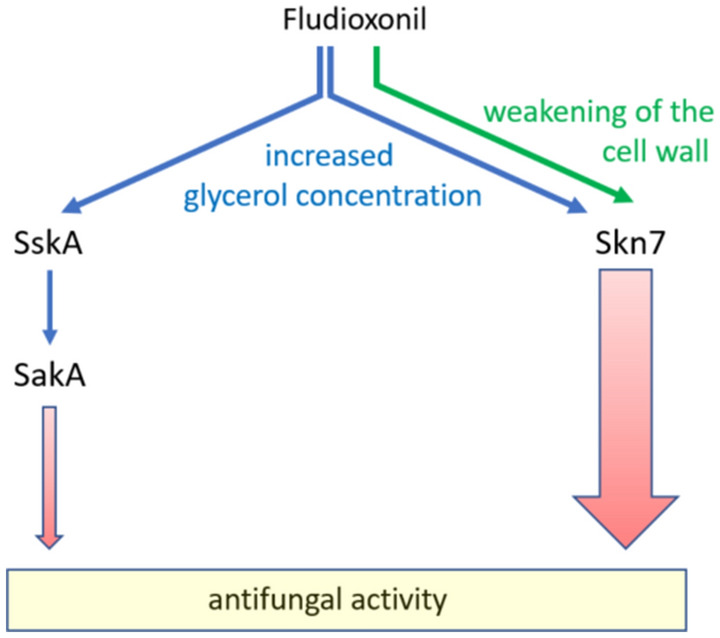
Schematic model of the proposed mechanisms mediating the antifungal activity of fludioxonil in *A. fumigatus.* Blue arrows: increase in the internal glycerol concentration. Green arrow: Weakening of the cell wall. Red arrows: The relative contribution of Skn7 and the SskA-SakA axis to antifungal activity of fludioxonil is indicated by the width of the respective arrow.

In conclusion, we have identified Skn7 as a key player in the antifungal activity of fludioxonil. The dramatic swelling of *A. fumigatus* cells is clearly dependent on the force that is provided by the increased internal osmotic pressure, but fludioxonil must trigger another, decisive process and data obtained in the current study hint towards a Skn7-mediated destabilization of the cell wall. This hypothesis is depicted in the schematic model shown in Fig. 9. Further work is underway to characterize this process in more detail.

## Methods

### Strains and media

Strain AfS35 is a nonhomologous end-joining-deficient derivative of the clinical *A. fumigatus* isolate D141^29^. The following strains were also used: *A. fumigatus* ATCC46645 and Af293 as well as *A. nidulans* R21, FGSC A26 and FGSC A4. The ∆*tcs*C mutant of AfS35 was generated and characterized by McCormick et al.^4^. The isolation of resting conidia and the Aspergillus Minimal Medium (AMM) had been previously described^30^.

### Genetic manipulations

The oligonucleotides used in this study are summarized in Suppl. Table 1. Q5 High Fidelity polymerase (New England Biolabs) was used in all cloning experiments. To generate deletion cassettes, approx. 1000 bp regions flanking the target gene were amplified using the oligonucleotide combinations specified in Suppl. Table 1. The PCR products were digested with SfiI and ligated to a 4.8 kb hygromycin B resistance cassette excised from plasmid pSK528 by SfiI digestion. The resulting deletion cassette was introduced into the recipient strain by protoplast transformation. To further verify the hygromycin-resistant mutants, sequences of approx. 700 bp in the different genes were amplified from the chromosomal DNA of the respective strain. To generate a double deletion mutant, the hygromycin B-resistance cassette of ∆*skn*7 was removed by growing the strain on medium containing xylose as sole carbon source^31^. The *sak*A gene was then deleted in the hygromycin-sensitive ∆*skn*7 mutant as described above.

For complementation of the ∆*sak*A strain, the *sak*A gene was amplified by PCR and cloned into the PmeI site of pSK379. For complementation of the ∆*skn*7 strain, pSK379 was first digested with PstI and PmeI to remove the *gpd*A promoter. In the next step, the *skn*7 gene and its promoter region were amplified using oligonucleotides Skn7-Pro-PstI-FOR and Skn7-REV. The *skn*7 PCR product was digested with PstI and the resulting fragment was ligated into the truncated pSK379 vector. The complementation plasmids were finally introduced into the respective mutants by protoplast transformation.

### Phenotypic characterization

Freshly isolated conidia were counted using a Neubauer improved chamber and series of ten-fold dilutions starting with 10^7^ conidia per ml were spotted onto AMM plates in aliquots of 1 μl. Plates supplemented with the indicated agents were incubated at 37 °C. For disk diffusion assays, AMM plates were homogenously inoculated with a swab from a suspension of the desired strain containing 10^7^ resting conidia per ml, paper disks were placed on these plates and loaded with the indicated amounts of effector molecules. For osmoprotection assays, AMM plates were supplemented with 1.2 M or 2 M sorbitol. Inhibition zones were measured using a Scan 4000 Ultra-HD colony counter (Interscience, Saint Nom, France). To obtain images, plates were usually placed on a black background, except for the caspofungin experiment, in which plates were placed on a light box.

### Determination of minimal inhibitory concentrations

Serial 1:2 dilutions of a fludioxonil stock solution in ethanol (2 mg/ml) were prepared in 100 µl AMM per well. Another 100 µl AMM containing 4 × 10^4^ conidia of the tested strains were added per well resulting in a total volume of 200 µl AMM per well. The final concentrations of fludioxonil in the assay ranged from 0.02 to 20 µg/ml. The highest concentration of ethanol applied in the fludioxonil test wells was used as a solvent control. Four parallel wells were prepared for each condition. After incubation for 72 h at 37 °C, the minimal inhibitory concentration for each strain was determined by visual and microscopic analysis of the individual wells. The experiment was repeated on the next day under identical conditions.

### Microscopic images

Glass coverslips were placed in the wells of a 24 well plate. One ml AMM plus 2 µg/ml fludioxonil were added per well and 5 × 10^5^ resting conidia of the desired strain. The cultures were incubated at 37 °C for 18 h. For bright field images, the coverslips were placed on a microscopic slide and micrographs were immediately taken using a Leica DM5000B equipped with a Leica DFC3000 G CCD camera (Leica Microsystems). For chitin stainings, samples were fixed with 3.7% formaldehyde in PBS for 5 min and stained with 100 µg/ml CFW for 5 min. Samples were mounted with Vectashield mounting medium (Vector Laboratories) and analyzed using an LSM 880 confocal laser scanning microscope (ZEISS, Germany). All microscopic images were taken using a 63 × objective. The images were recorded using the ZEN black Software, which is provided by ZEISS together with the confocal microscope. Adobe Photoshop CS6 Extended was used to combine the original images into multi-panel figures.

### Determination of the intracellular glycerol content

Conidia of the respective *A. fumigatus* strains were inoculated at a density of 7.5 × 10^5^/ml in 40 ml AMM and grown for 16 h at 30 °C and 150 rpm. Afterwards fludioxonil was added at a final concentration of 1 µg/ml and cultures where incubated for another 6 h at 37 °C and 160 rpm. The mycelium was harvested by filtration through Miracloth filter and stored at -80 °C. For glycerol extraction, 1050 µl PBS were added to 50 mg biomass in Lysing Matrix A tubes (MP Biomedicals) and were subsequently grinded using a Fast Prep 24 5G beating system (MP Biomedicals) for 40 sec at 6 m/sec. The cell debris was removed by centrifugation and the protein concentrations of the supernatants were determined using the Bio-Rad Protein Assay according to the instructions of the vendor (BioRad Laboratories). All samples were adjusted to a protein concentration of 100 µg/ml using PBS and the glycerol concentrations of these solutions were measured enzymatically using the Glycerol Assay Kit according to manufacturer’s protocol (Sigma-Aldrich). All experiments were performed in three parallel experiments (biological replicates) with at least two technical replicates each.

## Supplementary Information

Supplementary Information
