# Supplementary material for: The response regulator Skn7 of Aspergillus fumigatus is essential for the antifungal effect of fludioxonil
Source: Sci Rep. 2021 Mar 5;11:5317. doi: 10.1038/s41598-021-84740-6 (PMC7935864; doi:10.1038/s41598-021-84740-6)
Supplement: Supplementary file 1 — Supplementary Information [file 41598_2021_84740_MOESM1_ESM.pdf]

# - Supplementary Files -

The response regulator Skn7 of *Aspergillus fumigatus* is essential for the antifungal effect of fludioxonil.

Sebastian Schruefer[1#], Isabella Böhmer[1#], Karl Dichtl[2], Anja Spadinger[1], Christoph Kleinemeier[1], Frank Ebel[1]

1: Institute for Infectious Diseases and Zoonoses, Faculty of Veterinary Medicine, LMU Munich, Germany

2: Max von Pettenkofer Institute, Faculty of Medicine, LMU Munich, Germany

Supplementary Figure 1:

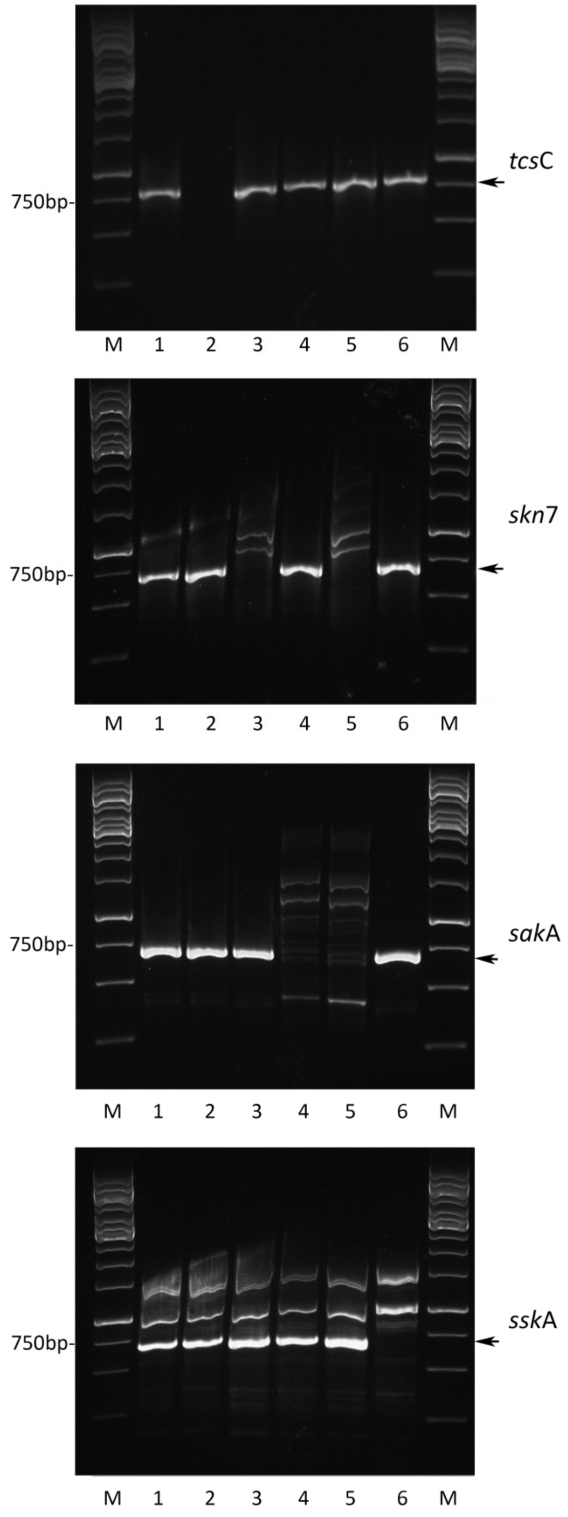

PCR analysis of the different strains used in this study. We have amplified fragments of approximately 700 bp of the genes indicated on the right margin from chromosomal DNA of the different strains using oligonucleotides that are specified in Suppl. Table 1. M = marker; 1 = AfS35; 2 = Δ*tcs*C; 3 = Δ*skn*7; 4 = Δ*sak*A, 5 = Δ*skn*7 Δ*sak*A; 6 = Δ*ssk*A. The specific amplicons are indicated by arrows.

Supplementary Figure 2:

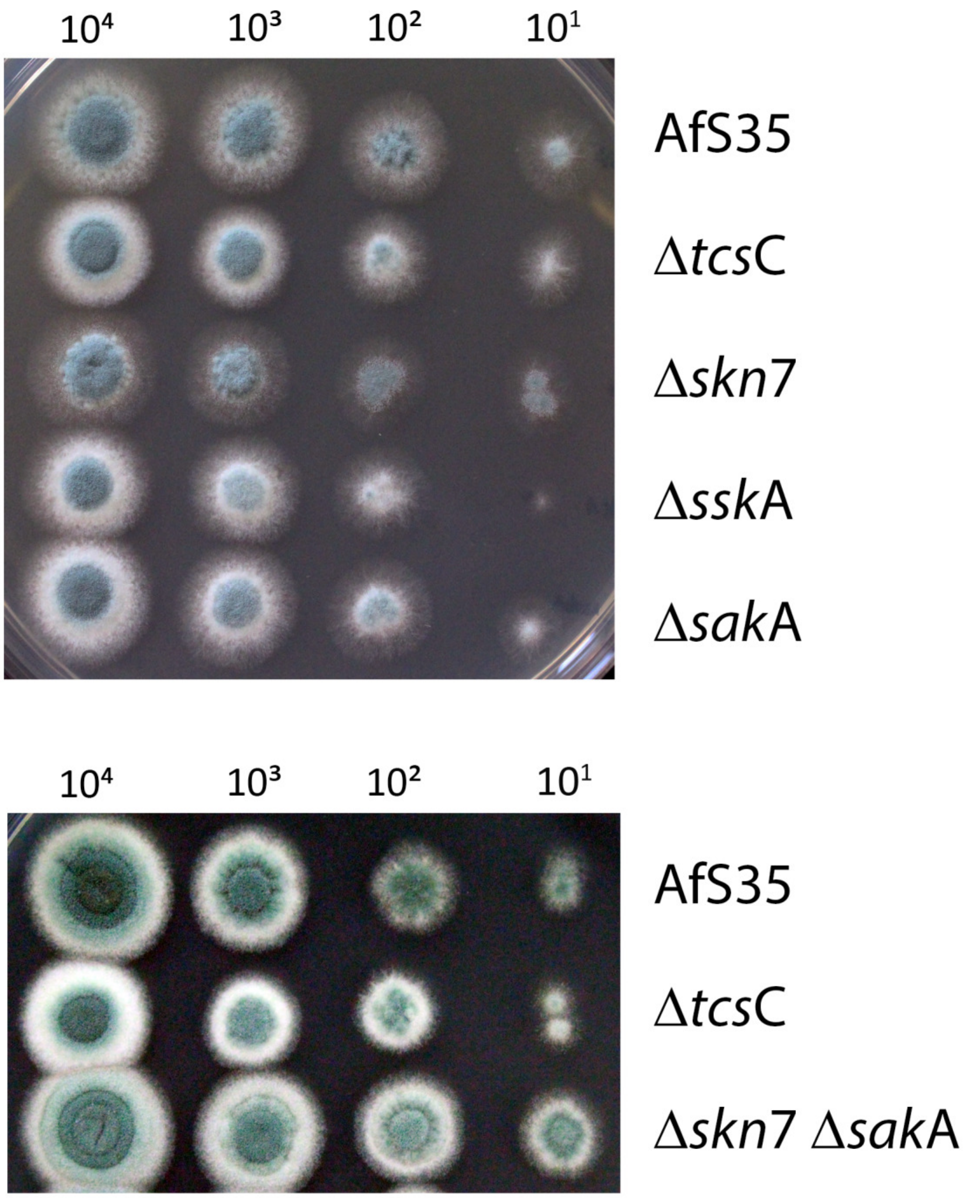

Growth of the *A. fumigatus* HOG pathway mutants on AMM agar. The drop dilution assays were incubated at 37°C for 48h. The strains and the number of conidia that were applied per drop are indicated.

Supplementary Figure 3:

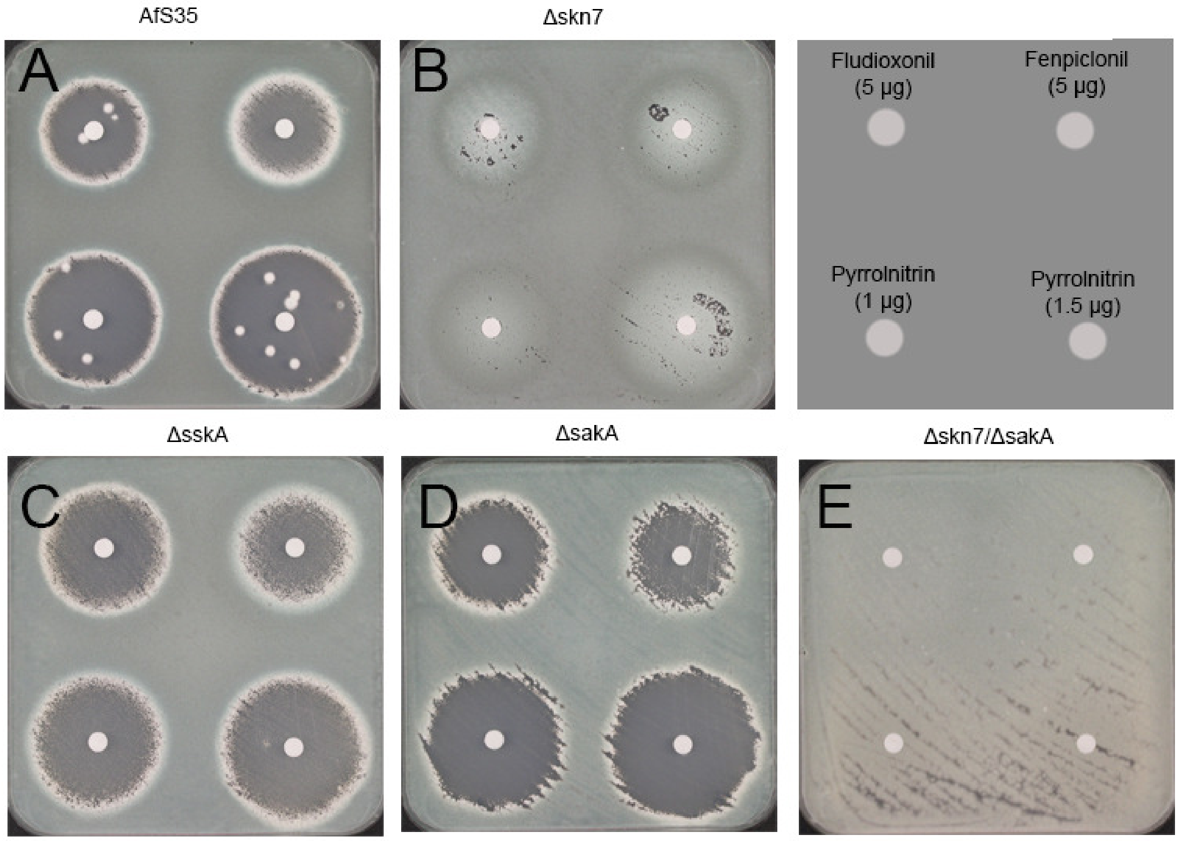

Comparison of the sensitivities of the indicated mutants to fludioxonil, fenpiclonil and pyrrolnitrin. Paper disks containing 5 µg fludioxonil, 5 µg fenpiclonil and 1 and 1.5 µg pyrrolnitrin were placed on plates that were inoculated with conidia of the following strainsd: AfS35 (A), Δ*skn*7 (B), Δ*ssk*A (C), Δ*sak*A (D) and Δ*skn*7 Δ*sak*A (E). The plates were incubated at 37°C and images were taken after for 48h.

Supplementary Figure 4:

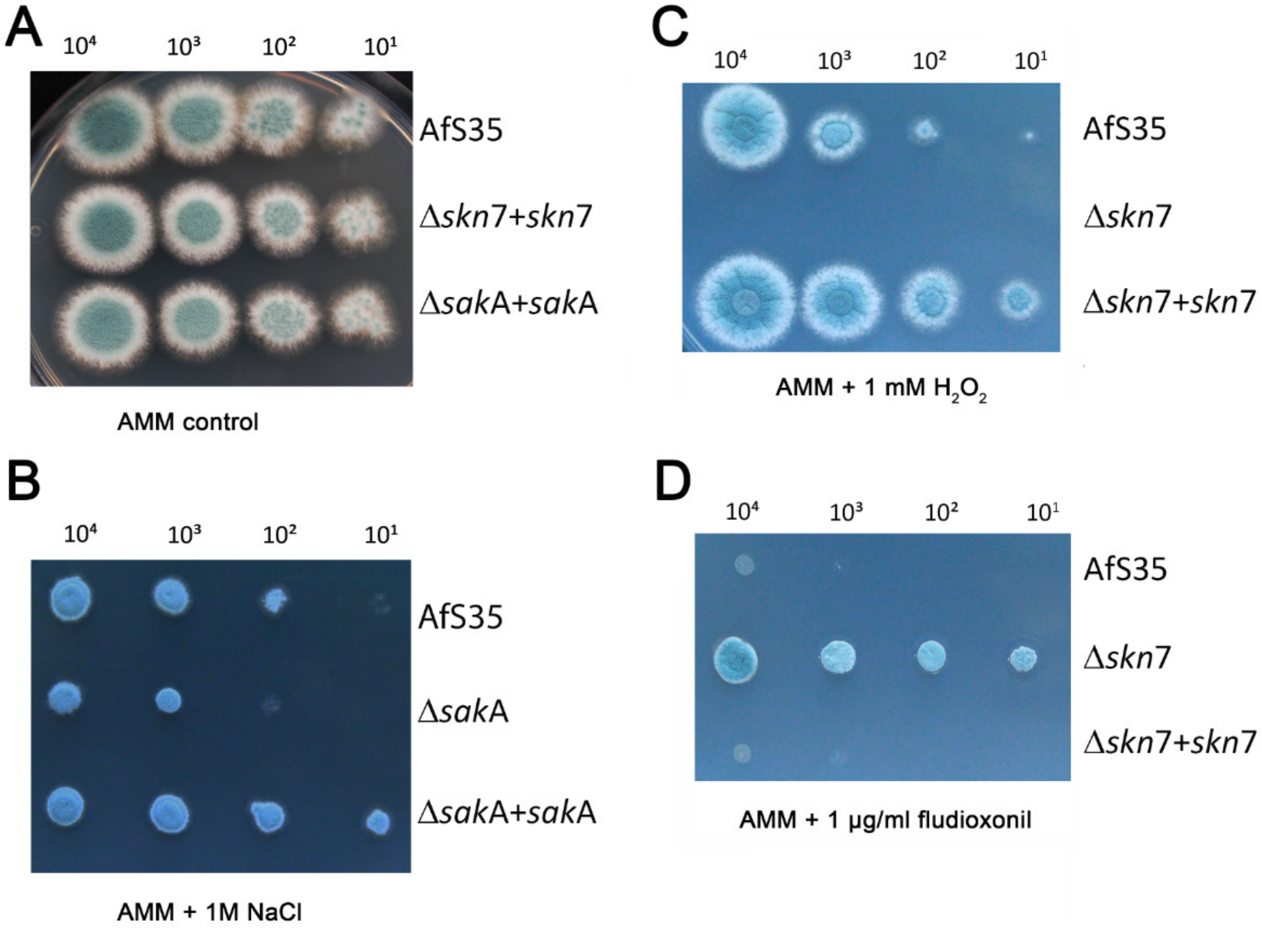

Functional complementation of the Δ*sak*A and the Δ*skn*7 mutant. Panel A: Drop dilution assay on an AMM plate without supplements demonstrating a normal growth for the two complemented mutants. Panel B: The Δ*sak*A+*sak*A strain shows a wild type-like resistance to high salt stress on AMM containing 1 M NaCl. Panels C and D: Drop dilution assays on AMM plates containing 1 mM H_2O_2 (C) or 1 µg/ml fludioxonil demonstrate the functional complementation of the Δ*skn*7 mutant (D).

Supplementary Figure 5:

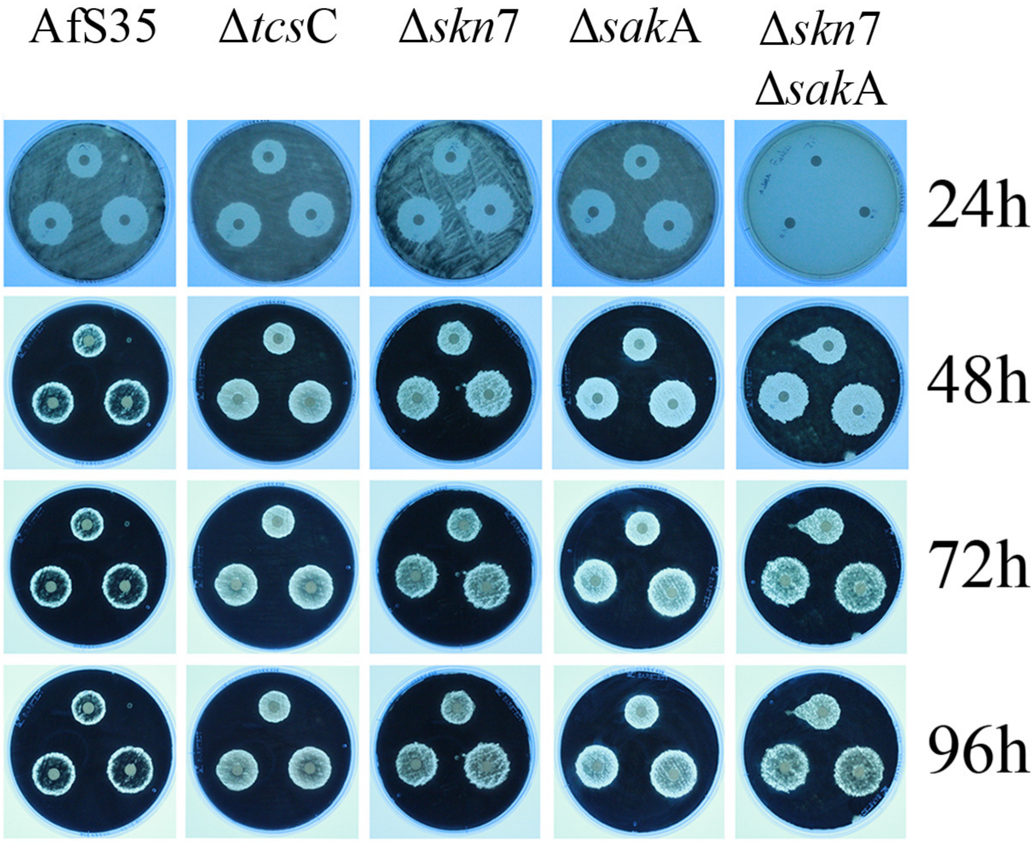

The HOG mutants show a reduced trailing growth on plates with paper disks containing 2.5 µg (top), 10 µg (left) or 20 µg (right) caspofungin. The AMM plates were inoculated with resting conidia and incubated at 37°C for the times indicated. Note that the trailing growth in the inhibition zones is most prominent for the wild type strain AfS35. This is also the only strain that sporulated well in the inhibition zone.

Supplementary Figure 6:

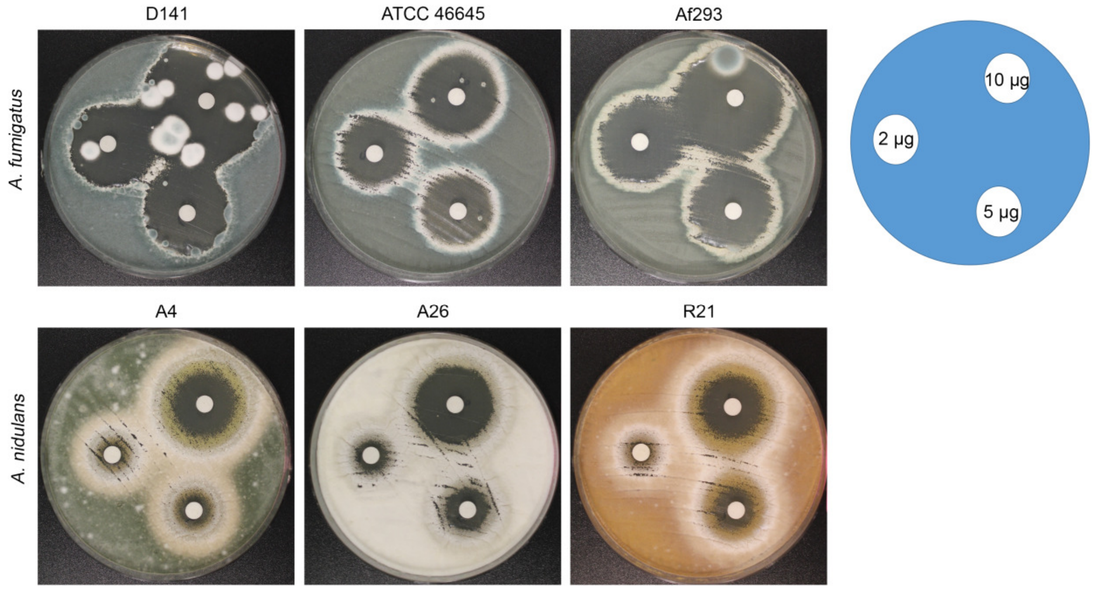

A. fumigatus strains are more susceptible to fludioxonil than *A. nidulans* strains. Sabouraud plates were inoculated with resting conidia of the *A. fumigatus* strains D141, ATCC 46645 and Af293 or the *A. nidulans* strains R21, A26 and A4. Paper disks with the indicated amounts of fludioxonil were then placed on the plates and images were taken after an incubation of 48h at 37°C.

Supplementary Table 1: Oligos used in this study

Designation	Sequence	Experiment
SakA-1000bp-up-FOR	TTGATTTCTCCTCTAAGCCCG	deletion of *sak*A up-stream
SakA-1000up-REV-SfiI	GCGGCCTGAGTGGCCTTTGGATAGTGTGGGTGG	deletion of *sak*A up-stream
SakA-1000do-FOR-SfiI	GCGGCCATCTAGGCCAAGTGGTCACCATGTGCA	deletion of *sak*A down-stream
SakA-1000bp-do-REV	AACACGATACAATGGGGTCTC	deletion of *sak*A down-stream
SakA-FOR	ATGGCCGAGTTCGTGCGT	complementation of Δ*sak*A
SakA-REV	TTATGCATAGTTTTGTTG	complementation of Δ*sak*A
SskA-1000bp-up-FOR	ATGTTTTTTCAGAGAGCGCCA	deletion of *ssk*A up-stream
SskA-1000up-REV-SfiI	GCGGCCTGAGTGGCCGATGAGGATCCACCACAG	deletion of *ssk*A up-stream
SskA-1000do-FOR-SfiI	GCGGCCATCTAGGCCCCAGTTGCACTTTCTGCA	deletion of *ssk*A down-stream
SskA-1000bp-do-REV	AACGCAAGAGACTCGCCAAGG	deletion of *ssk*A down-stream
Skn7-1000up-For	GCGTTAGGACTTGGGACC	deletion of *skn*7 up-stream
Skn7-1000up-REV-SfiI	GCGGCCTGAGTGGCCCGTGGGCTAGATGGG	deletion of *skn*7 up-stream
Skn7-1000do-FOR-SfiI	GTGGCCATCTAGGCCGGTGAGAACAGTCGA	deletion of *skn*7 down-stream
Skn7-1000do-REV	ACCTCGGGCGGTCAGCGA	deletion of *skn*7 down-stream
Skn7-ATG-FOR	ATGGAGGGTGGCCAGACC	complementation of Δ*skn1*
Skn7-REV	TTAGCCACTTCGAGTAGC	complementation of Δ*skn1* P_{gpdA}
Skn7-Pro-PstI-FOR	GACTGCAGCTGAGGACGATCATAATGCA	complementation of Δ*skn1* P_{skn7}
SakA-FOR	ATGGCCGAGTTCGTGCGT	PCR detection of *sak*A
SakA-700-REV	AATACATGGTTAGCGTTC	PCR detection of *sak*A
SskA-FOR	ATGCCTGACCGCCGCCTG	PCR detection of *ssk*A
SskA-700-REV	GCACGCTGGAATTTTCTC	PCR detection of *ssk*A
Skn7-ATG-FOR	ATGGAGGGTGGCCAGACC	PCR detection of *skn*7
Skn7-700-REV	CTTGCTGTTCGCTCTGAA	PCR detection of *skn*7
TcsC-His-FOR	GCGAATTCAACCTATGATTTCAAATAC	PCR detection of *tcs*C
Tco1-REV	TTCTCATACGGCCTTTGGAGAGCG	PCR detection of *tcs*C
